# Early Molecular Responses of Tomato to Combined Moderate Water Stress and Tomato Red Spider Mite *Tetranychus evansi* Attack

**DOI:** 10.3390/plants9091131

**Published:** 2020-08-31

**Authors:** Vicent Arbona, Miguel G. Ximénez-Embún, Alberto Echavarri-Muñoz, Marcos Martin-Sánchez, Aurelio Gómez-Cadenas, Félix Ortego, Miguel González-Guzmán

**Affiliations:** 1 Departament de Ciències Agràries i del Medi Natural, Universitat Jaume I, 12071 Castelló de la Plana, Spain; arbona@uji.es (V.A.); aurelio.gomez@uji.es (A.G.-C.); 2 Departamento de Biotecnología Microbiana y de Plantas, Centro de Investigaciones Biológicas Margarita Salas, CSIC, 28040 Madrid, Spain; miguelgxe@hotmail.com (M.G.X.-E.); albertoech@hotmail.es (A.E.-M.); martinsanchezmarcos@gmail.com (M.M.-S.); ortego@cib.csic.es (F.O.)

**Keywords:** plant-herbivore interaction, drought stress, spider mites, tomato, combined stresses

## Abstract

Interaction between plants and their environment is changing as a consequence of the climate change and global warming, increasing the performance and dispersal of some pest species which become invasive species. *Tetranychus evansi* also known as the tomato red spider mite, is an invasive species which has been reported to increase its performance when feeding in the tomato cultivar Moneymaker (MM) under water deficit conditions. In order to clarify the underlying molecular events involved, we examined early plant molecular changes occurring on MM during *T. evansi* infestation alone or in combination with moderate drought stress. Hormonal profiling of MM plants showed an increase in abscisic acid (ABA) levels in drought-stressed plants while salicylic acid (SA) levels were higher in drought-stressed plants infested with *T. evansi*, indicating that SA is involved in the regulation of plant responses to this stress combination. Changes in the expression of ABA-dependent *DREB2*, *NCED1*, and *RAB18* genes confirmed the presence of drought-dependent molecular responses in tomato plants and indicated that these responses could be modulated by the tomato red spider mite. Tomato metabolic profiling identified 42 differentially altered compounds produced by *T. evansi* attack, moderate drought stress, and/or their combination, reinforcing the idea of putative manipulation of tomato plant responses by tomato red spider mite. Altogether, these results indicate that the tomato red spider mite acts modulating plant responses to moderate drought stress by interfering with the ABA and SA hormonal responses, providing new insights into the early events occurring on plant biotic and abiotic stress interaction.

## 1. Introduction

Plant life is continually challenged by different environmental stresses which must be endured by making physiological and metabolic adjustment to continue their life cycle. However, climate change and global warming are modifying the interaction between plants and their environment. In most cases, plants are going to suffer more rigorous abiotic and biotic stresses which will produce huge losses in yield and fruit quality in a context characterized by a growing world population [1,2]. At the same time, the interaction between plants and several phytophagous pests are also going to change because worldwide temperature increment is expanding pest/pathogen distribution and incidence, and subsequent crop damage [3]. To face single or combined stresses, plants must synchronize the activation of a set of plant response genes to produce biochemical and metabolic changes, which will allow plant adaptation to the new and specific environmental conditions [4,5]. This genetic reprogramming is achieved through the coordinated action of several phytohormones which may act in different phases of the adaptation and response to the stress combination [5,6].

Tomato (*Solanum lycopersicum* L. Mill) is one of the most important horticultural crops worldwide with a production of 182,000 millions of tons with an economic value higher than $65 billion during the 2018 year (FAOSTAT database, http://www.fao.org/faostat/en/#data). Most of the current commercial tomato cultivars are sensitive to biotic and abiotic stresses that impact tomato productivity and quality, affecting seed development and germination, vegetative growth, and fruit growth and ripening [7]. Several species of spider mites belonging to the Tetranychidae family produce huge crop damage in a short time, owing to their high reproductive potential, a feeding mechanism that allows mites to suck plant mesophyll cell content and a high ability to develop resistance to acaricides [8,9]. Among them, the two-spotted spider mite *Tetranychus urticae* is a worldwide extended member because of its polyphagous nature, feeding on over 1000 different plants species [10]. However, some plant specialist species such as the tomato red spider mite, *Tetranychus evansi* Baker & Pritchard, native to South America have become an emerging pest of solanaceous crops since it is spreading over the world due to the increase in worldwide temperatures. In the last decades, *T. evansi* has expanded its geographical distribution in many regions of North America, Africa, Europe, and East Asia [11].

Plant defence mechanisms against spider mites include physical constitutive barriers such as trichomes as well as induced defences which produce a large variety of secondary metabolites including polyphenols, phenylpropanoids, or isoprenoids that act as chemical weapons or arthropod predator attractants [12,13,14]. Transcriptomic studies have confirmed that plant defences against spider mites are mainly steered by jasmonates (JAs) and salicylic acid (SA), but also point to the involvement of other plant hormones such as abscisic acid (ABA) and ethylene (ET) which fine-tune plant defence responses according with environmental conditions [15,16,17]. Proteomics and metabolomics studies have identified putative protein effectors coming from mite feeding that may be recognized by the plant defence system to initiate a successful plant defence response or in other cases, used by the spider mite to interfere and supress the plant defence system. Several pioneering works identified (i) two salivary proteins named as effectors 28 and 84, which are present in *T. urticae* and *T. evansi*, have been involved in suppression of JA and SA-dependent responses, (ii) a family of mite secreted salivary proteins referred as SHOT, which are produced depending of the host identity, and (iii) two secreted proteins named tetranins which could upregulated plant defences [14,18,19,20,21]. Moreover, spider mites may induce defence responses in systemic leaves and some strains and species of mites as *T. evansi* are able to suppress or reduce tomato defence responses [17,22,23], uncovering the complexity of the plant–spider mite interaction.

Several analyses of *S. lycopersium*-*T. evansi* interaction have shown that the tomato red spider mite is able to suppress tomato anti-mite defenses, increasing mite fitness and rendering tomato plants more vulnerable to other spider mite species [22,24]. At the hormonal level, these genetic analyses have concluded that *T. evansi* suppresses or attenuates the expression of inducible JAs and SA-dependent defense genes, indicating that the suppression of plant defenses occurs at the level of hormonal signaling pathways and the reduced hormone accumulation is a consequence of the altered feedback regulation, although the mechanisms involved are still poorly understood [17,22,23]. Moreover, the recent analysis by Schimmel et al. [17] showed that *T. evansi* infestation modified the expression of only 38 genes of the 16,431 tomato genes analyzed, in contrast with the 2460 tomato genes that modified their expression by *T. urticae* infestation, suggesting the occurrence of a reduced number of physiological changes in tomato red spider mite infested plants.

On the other hand, abiotic stresses like drought stress enable plant adaptive responses which are controlled by a complex phytohormonal network where ABA plays a key role [25,26,27]. However, ABA has also been found to be involved in stress responses to pathogens, ranging its action from promoting resistance by inhibiting pathogen entry via stomata to increasing plant susceptibility by interfering with defence responses mediated by other signaling pathways [13,28]. Moreover, it has been described that physiological and metabolic changes produced during the plant adaptation to drought stress can increase phytophagous arthropod infestation by reducing the plant defence mechanisms or/and by increasing the nutritional value of the plant since many plant osmolytes that accumulate under drought stress are sugars and amino acids such as the disaccharide trehalose or the amino acid proline [25,29]. However, this interaction has been shown to be more complex since it also depends on the plant species, the intensity and duration of the drought stress and the arthropod feeding mechanism [30,31]. Previous results coming from the analysis of the interaction between *T. urticae* or *T. evansi* and moderate water stress conditions in tomato plants (cv Moneymaker) showed the increase of both spider mite populations, thus confirming the modification of the plant–spider mite interaction under water stress conditions [21,32,33]. Moreover, the characterization of the *T. evansi* attack to traditional drought-tolerant tomato varieties has shown that adaptation of tomato plants to moderate drought modifies physiological plant responses to tomato red spider mite associated with an increase of tomato ABA levels [21]. All these previous results indicate that plant response mechanisms boosting *T. evansi* growth under drought stress conditions and those by which it supresses plant defences might be independent, and that ABA is involved in the fine-tune of spider mite-mediated plant defences [21,32].

In the present study, we aim to identify early physiological, hormonal, and metabolic changes involved in the tomato red spider mite interaction under moderate water deficit conditions, addressing which plant key factors and molecular mechanisms may be involved in this abiotic–biotic stress interaction. We have analyzed (1) plant physiological parameters as leaf stomatal conductance, plant growth, and photosynthetic efficiency, (2) endogenous hormonal levels of key hormones such as ABA, SA, and JAs, (3) the transcription pattern of some biotic and abiotic stress-dependent genes and (4) the metabolite profile of *S. lycopersicum* cv Moneymaker under moderate water stress conditions, tomato red spider mite *T. evansi* attack, and a combination of both.

## 2. Results

### 2.1. Physiological Characterization of Tomato Plants under Moderate Drought and T. evansi Stress Combination

**Figure 1:**
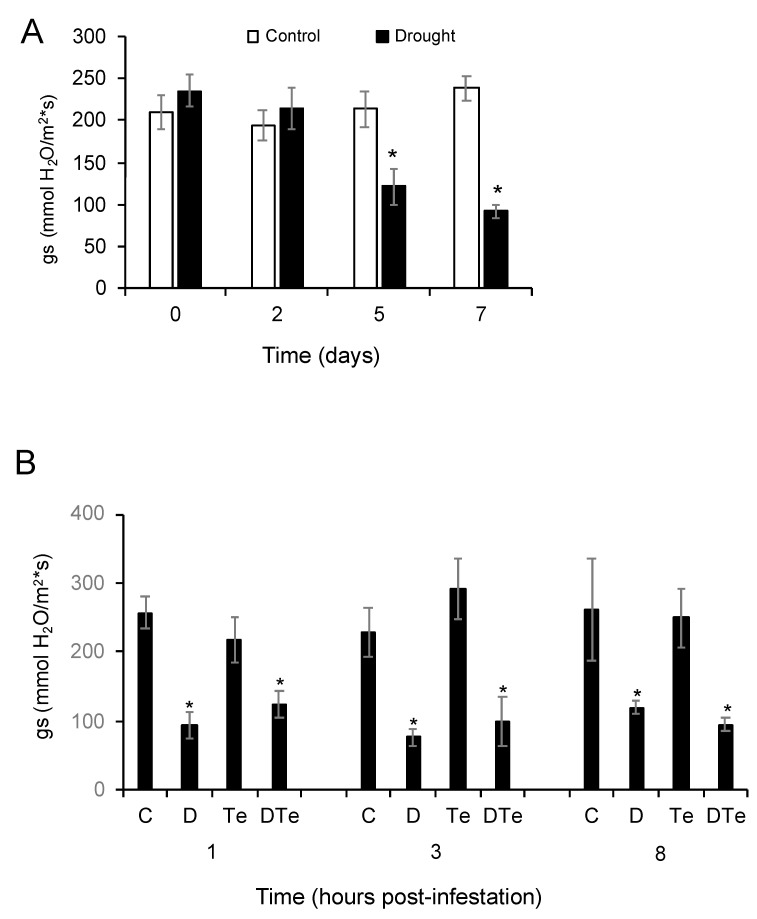
Characterization of leaf stomatal conductance (gs) during tomato red spider mite and moderate drought experiments. (**A**) gs values during the period of drought stress imposition in control plants (white) and moderate drought stressed plants (black), (**B**) gs values on uninfested well-watered control plants—C; uninfested moderate drought-stressed plants—D; infested well-watered plants—Te; and infested moderate drought-stressed plants—DTe at one-, three-, and eight hours post infestation (hpi) with tomato red spider mites. Data are mean ± SE of seven replicates/treatment from two independent experiments. Asterisk indicates significant differences of each treatment respect its control (Student’s *t*-test, *p* < 0.05).

One of the most established plant responses during a drought stress period is the reduction of plant stomatal conductance to avoid water loss, which leads to a decrease in the photosynthetic activity and the progressive arrest of plant growth when drought stress becomes severe [25]. As a part of the characterization of moderate drought stress conditions in tomato cv. Moneymaker after water withdrawal and prior to plant mite infestation, we monitored the saturation weight content (SW) [SW = (weight of 100% water saturated soil − soil weight)/weight of 100% water saturated soil], plant stem growth, photosynthetic efficiency, and leaf stomatal conductance (gs) at two, five, and seven days after water withdrawal. After seven days of withholding watering, tomato plants reached moderate water stress conditions with a SW value of ~45% and a gs value that was 33% of that in control plants (Figure S1A and Figure 1A). No significant differences were observed in stem growth or photosynthetic activity between control and drought stressed plants during this period (Figure S1B,C). In addition, gs was also analyzed when plants were subjected to moderate drought stress, tomato red spider mite infestation, and their combination. No significant differences in gs values were observed between control and *T. evansi* infested plants at any time point, whereas a significant reduction of gs was found in plants under moderate drought stress alone or in combination (Figure 1B), indicating that tomato red spider mite infestation does not affect tomato leaf stomatal conductance at early infestation stages as reported for longer infestation times [32].

### 2.2. Hormonal Levels of Moderate Drought-Stressed Tomato Plants Alone or Combination with T. evansi

**Figure 2:**
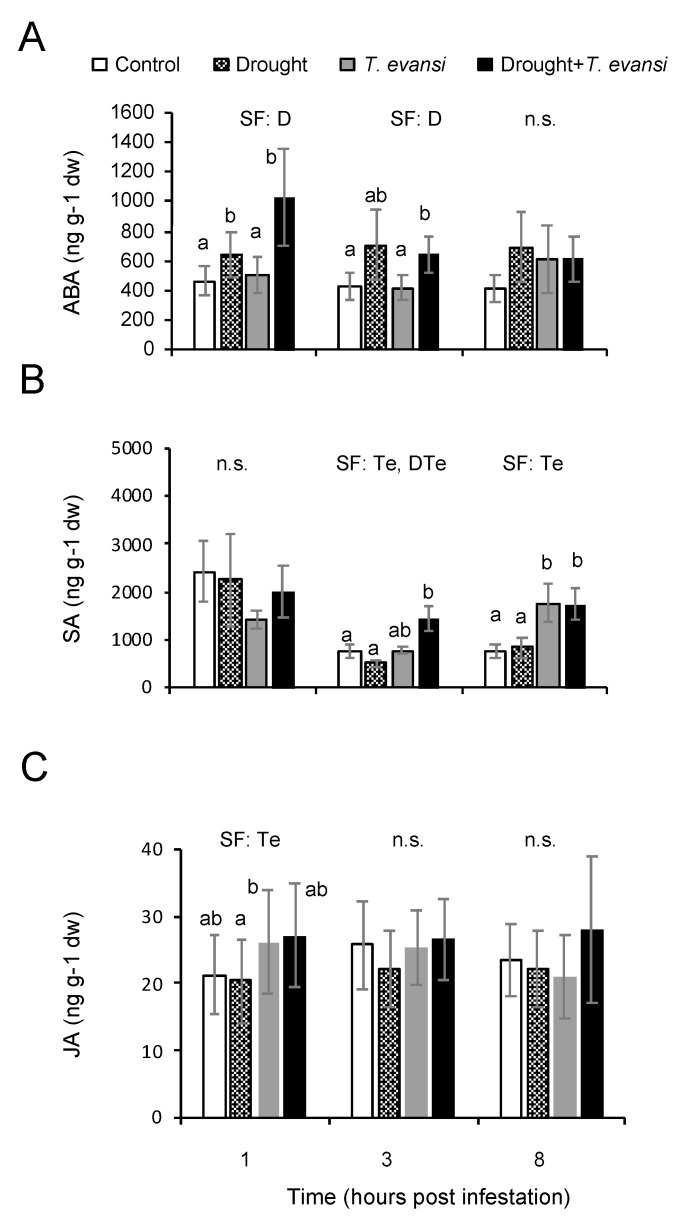
Hormone profiling of tomato plants under moderate water stress and tomato red spider mite infestation. (**A**) ABA, (**B**) SA, and (**C**) JA endogenous levels in uninfested well-watered plants (Control); uninfested moderate drought-stressed plants (Drought); infested well-watered plants (*T. evansi*); and infested moderate drought-stressed plants (Drought + *T. evansi*). D (moderate drought condition), Te (*T. evansi* infestation) and their combination DTe (Drought + *T. evansi*) factors were analyzed by two-way ANOVA (*p* < 0.05), including the experiment replica as a fixed factor. Significant contributing factors (SF) are indicated. n.s. indicates that no significant factor was found. When a treatment factor was significant, the Tukey HSD post hoc test was performed (different lowercase letters indicate significant differences among treatments). The detailed results of the two-way ANOVA (F and *p* values and degrees of freedom) are shown in Table S2A.

In order to characterize the hormonal dynamics controlling plant responses to moderate drought and spider mite infestation alone or in combination, the hormonal levels of the most important stress hormones such as ABA, JA, and SA were determined during the seven days of moderate water stress imposition (Figure S2 and Table S1A) and in plants under drought and tomato red spider mite infestation alone or in combination at one-, three-, and eight-hpi (Figure 2 and Table S2A). A significant increase in ABA levels was identified in tomato plants under moderate water stress at five and seven days after stress imposition and in plants at one- and three-hours after stress combination, indicating that tomato plants were responding to the moderate water stress conditions at the hormonal level (Figure 2A and Figure S2A). No significative differences in SA and JA hormonal levels were found during the seven days of moderate water stress imposition (Figure S2B,C). However, SA hormonal levels were higher in tomato plants infested with tomato red spider mites at three- and eight-hpi than in control plants (Figure 2B). The ANOVA analysis showed that the tomato red spider mite factor was significant at three- and eight-hpi while the stress combination factor was significant at three hours (*p* < 0.05, two-way ANOVA test), indicating that the increase of SA levels by the tomato red spider mites attack alone or in combination with moderate stress will putatively produce a differential SA signaling response. Interestingly, in control plants, we could observe an overall reduction of ~30% of the SA levels at three- and eight-hpi respect to SA level at one-hpi, which may be related to the already described circadian clock-dependent oscillation of SA and JA as part of the plant immune response [34,35].

Only significant differences in endogenous JA levels were observed in plants at one-hpi (Figure 2C), suggesting that tomato plants could sense the onset of *T. evansi* infestation and initiate the JA-dependent defence response as has been described for *T. urticae* infestation. However, there is no difference in JA levels at three- and eight-hpi, which indicated that the *T. evansi* mechanism to suppress tomato plant defences is effective as early as three-hpi, blocking from this point any subsequent JA induction and later JA-dependent defences as previously described [16,17,22].

### 2.3. Stress Response Gene Expression Analysis of Tomato Plants

In order to gain knowledge of the genetic changes induced by ABA and SA on the molecular plant response to moderate drought and tomato red spider mite, alone or in combination, the transcription level of some stress-induced marker genes was assessed. These markers genes were: the ABA biosynthesis 9-cis-epoxycarotenoid dioxygenase 1 (*NCED1*) gene [36]; the transcription factor dehydration-responsive element-binding protein 2 (*DREB2*) [37] and responsive to ABA 18 (*RAB18*) [38] genes which are ABA signaling pathway elements involved in plant drought stress responses; the JA biosynthesis allene oxide cyclase (*AOC*) gene [39]; the transcription factor jasmonic acid 2 (*JA2*) gene which is a putative link between JA and ABA signaling pathways that control stomatal aperture during bacterial attack [40]; the *MYC2* gene which is the master regulator of the JA-dependent plant responses to biotic stresses and wounding [41]; and the pathogenesis-related protein 1a (*PR-1a*) gene which is involved in SA-dependent biotic stress responses [42].

First, we analyzed stress marker gene expression patterns during the seven days of moderate water stress imposition (Figure S3 and Table S1B. No significant changes were observed in *DREB2*, *NCED1*, *JA2*, *MYC2*, and *PR1-a* gene expression, while the *AOC* gene showed only a significant reduction of their expression in drought stressed plants at two days. Remarkably, the drought-induced *RAB18* gene showed a clear gene expression induction of 13 and 22- fold-increase in drought-stressed compared with control plants at five and seven days after the experiment onset, respectively (Figure S3B).

**Figure 3:**
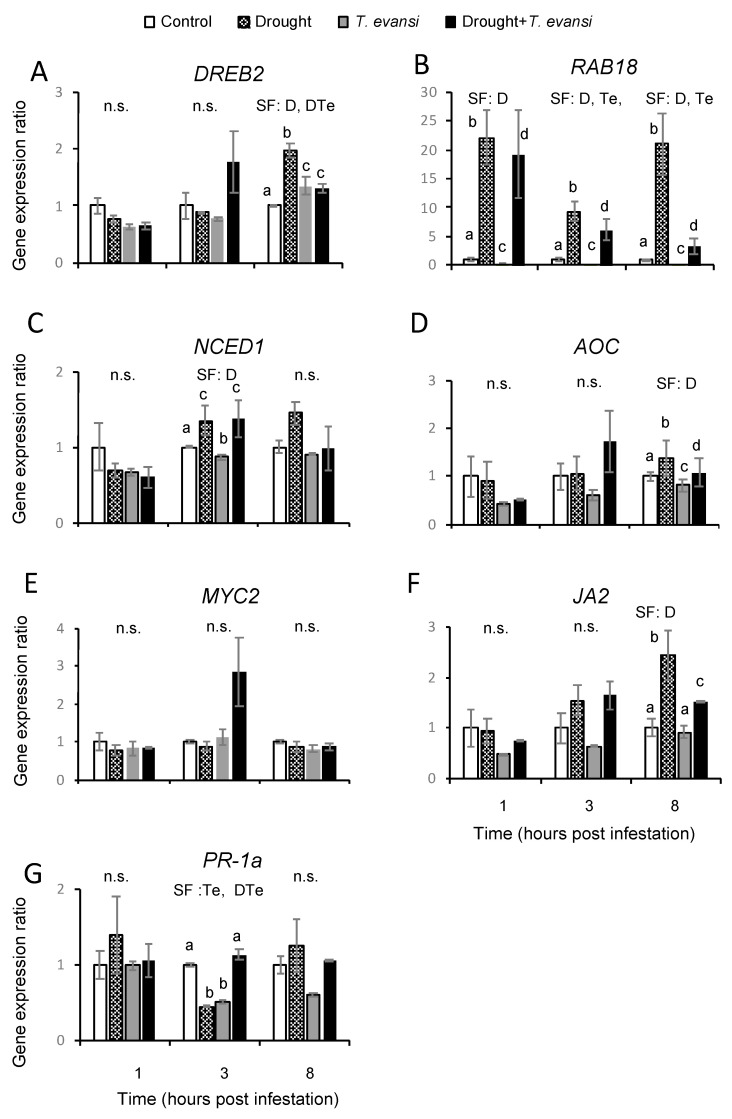
Stress-marker gene expression analysis of tomato plants under moderate drought stress and tomato red spider mite attack alone or in combination. The expression of (**A**) *DREB2*, (**B**) *RAB18*, (**C**) *NCED1*, (**D**) *AOC*, (**E**) *MYC2*, (**F**) *JA2*, and (**G**) *PR-1a* genes were analyzed at one-, three-, and eight-hours post infestation (hpi). Gene expression values are represented as gene expression ratio compared to its control sample for each time point. Data are mean ± SE of seven replicates/treatment from two experimental replicates: uninfested well-watered plants (Control); uninfested drought-stressed plants (Drought); infested well-watered plants (*T. evansi*); and infested drought-stressed plants (Drought + *T. evansi*). D (drought condition), Te (*T. evansi* infestation) and their combination DTe (Drought + *T. evansi*) factors were analyzed by two-way ANOVA (*p* < 0.05). Significant contributing factors (SF) are indicated. n.s. indicates that no significant factor was found. When a factor was significant, the Tukey HSD post hoc test was performed (different lowercase letters indicate significant differences among treatments). The detailed results of the two-way ANOVA (F and *p* values and degrees of freedom) are shown in Table S2B.

Next, we analyzed the gene expression pattern of the marker stress genes in plants under drought and tomato red spider mite infestation alone or in combination at one-, three-, and eight-hpi (Figure 3 and Table S2B). From all genes analyzed, the *RAB18* gene is the gene that showed more differences in its expression during treatments (Figure 3B). As expected, moderate drought stress treatment was a significant factor at all time points analyzed, reaching the *RAB18* gene expression a maximum of ~22-fold induction at one- and eight-hpi. Similar *RAB18* induction was observed in plants both infested and drought stressed at one- and three-hpi, but the stress combination factor was not significant. Interestingly, *RAB18* gene induction was clearly reduced in dual-stressed plants at eight-hpi, which indicates a differential response in plants under moderate water stressed alone or in combination with tomato red spider mites. The other two drought-marker genes, *NCED1* and *DREB2* genes, showed more specific differences. On one hand, drought was a significant factor for *NCED1* gene expression at three-hpi with similar values in drought-stressed plants alone or in combination with tomato red spider mites (Figure 3C). On the other hand, moderate drought and stress combination were significant factors for *DREB2* gene expression at eight-hpi, showing that the stress combination is modifying not only *RAB18* gene expression but also the *DREB2* drought-dependent gene expression (Figure 3A).

The gene expression of the *MYC2* gene, which is the master regulator of the JA-dependent response [41], did not show any significant variation during the time course (Figure 3E). However, the expression of *AOC* and *JA2* genes was significantly induced by moderate drought stress treatment at eight-hpi (Figure 3D,F). The gene expression of the SA-dependent *PR-1a* gene was significantly different in *T. evansi* infested plants alone or in combination with moderate drought at three-hpi, with a reduced expression in plants subjected to either of the individual stresses but not in plants subjected to both stresses (Figure 3G).

In summary, expression patterns of stress marker genes confirmed that plant stress responses to moderate drought and spider mite infestation alone or in combination were different.

### 2.4. Metabolite Profiling

Previous identification of tomato metabolic changes to moderate water deficit by biochemical and targeted metabolite profiling have shown a significant variation in amino acids and sugar composition, which may increase the nutritional value of the tomato plants leading to increase the leaf damage and oviposition of *T. evansi* in tomato drought-stressed plants [21,32,33]. To increase knowledge on the plant metabolites produced during concurring *T. evansi* attack and moderate drought stress on tomato, a non-targeted metabolite profiling of semi-polar metabolites was conducted using UPLC/ESI-QTOF-MS in positive and negative ion modes.

**Figure 4:**
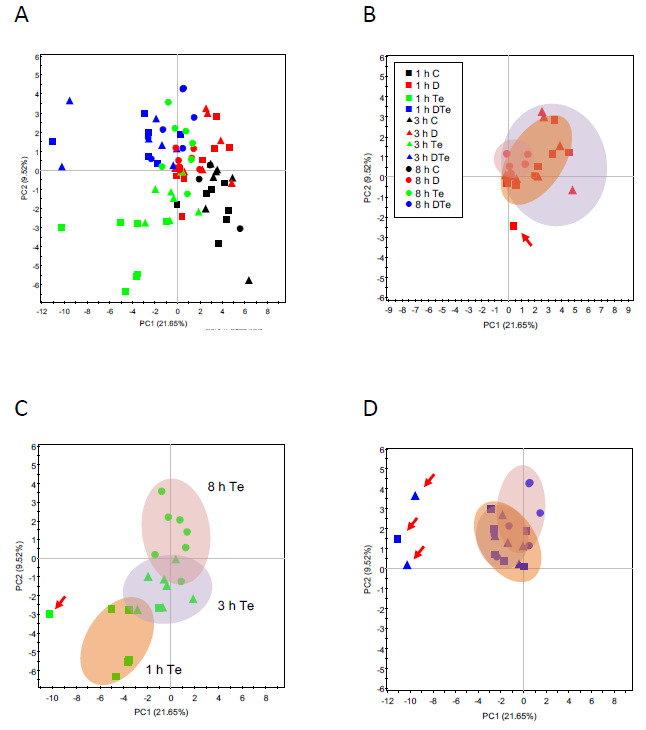
Principal Component Analysis (PCA) scores plots of PC1 vs. PC2 showing the spatial distribution of plant samples according to non-targeted metabolite profiling and hormonal data: (**A**) general view including all samples and sample groups, (**B**) moderate drought-stressed samples, (**C**) *T. evansi*-infested samples, and (**D**) moderate drought-stressed and *T. evansi*-infested samples. Outlier samples are highlighted with a red arrow. Well-watered control plants—C; uninfested moderate drought-stressed plants—D; infested well-watered plants—Te; and infested moderate drought-stressed plants—DTe.

**Figure 5:**
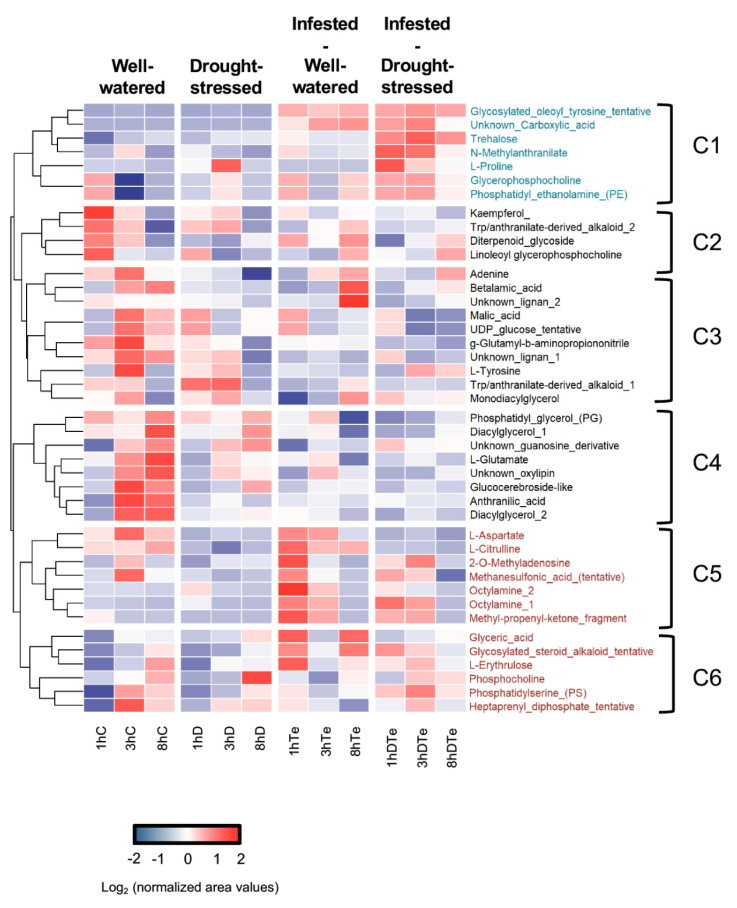
Heat map depicting significantly-altered metabolites in leaves of tomato in response to moderate drought—D, *T. evansi* infestation—Te and their combination—DTe at one-, three-, and eight-hours (h) post infestation. C accounts for uninfested well-watered control plants. Metabolites with relevant accumulation trends are highlighted in different colors and explained in the text. Data in cells are Log_2_-transformed mean values of seven biological replicates from two independent experiments.

Multivariate analysis of metabolomics data showed moderate differences in metabolite accumulation with a clear sample grouping (Figure 4A). The infestation of drought-stressed plants with *T. evansi* did not dramatically alter metabolite profiles respect to uninfested drought-stressed plants (Figure 4B,D), despite some differences could be spotted when analyzed in detail (Figure 5, Tables S3 and S4). Infestation with *T. evansi* induced a clear differentiation from control and moderate drought-stressed plants, also exhibiting clear differences among time points (Figure 4C). In summary, metabolite profiling analyses indicated that *T. evansi* attack induced significant metabolic changes at early time points, tending to a metabolic state with less differences with moderate drought-stressed and dual-stressed plant samples (Figure 4B–D). To the best of our knowledge, it is the first time that such significant metabolic changes at early mite infestation stages have been reported since it has been shown that *T. evansi* attack only produces 38 differentially expressed genes after seven days of infestation [17].

Metabolite profiling analyses revealed 1273 differentially expressed mass chromatographic features in response to drought and/or *T. evansi* infestation that were annotated as 42 individual metabolites and grouped into six trend clusters (Figure 5). Cluster C2, C3, and C4 comprised metabolites with higher levels in well-watered uninfested control plants followed by drought-stressed plants and much lower levels in well-watered and water-stressed mite-infested plants at all time points, with the exception of Adenine, Betalamic acid, and an unknown lignan which exhibited a sharp accumulation in *T. evansi* infested plants and, to a lower extent, in combined stress samples at eight-hpi. This cluster is formed by metabolites from diverse metabolic pathways such as the flavonoid kaempferol, aminoacids such as L-tyrosine and L-glutamate, phenylpropanoid precursors such as anthranilic acid several lipid derivatives such as two uncharacterized diacylglycerols, phospholipids such as phosphatidylglycerol, and linoleoyl glycerophosphocholine and a molecule similar to a glucocerebroside (Figure 5). Interestingly, no cluster showed any clear effect of drought on metabolite configuration of tomato plants.

Clusters C5 and C6 (highlighted in brown, Figure 5) included metabolites, of which accumulation was associated primarily to the *T. evansi* factor with clear differences between time points. All metabolites comprised within these clusters showed higher levels at one-hpi to decrease thereafter, and included compounds such as aminoacids L-aspartate and L-citrulline (by product of nitric oxide synthesis from L-arginine), nitrogen compounds such as 2-O-methyladenosine, glyceric acid, L-erythrulose, phosphatidylcholine, and phosphatidylserine, among others tentatively annotated such as geptaprenyl diphosphate, methanesulfonic acid (potentially derived from a sulfide-containing organic molecule), two octylamine moieties (probably derived from thiol-containing aliphatic molecules) and a methyl-propenyl ketone fragment (probably derived from volatile ketones) indicating that *T. evansi* infestation affected several pathways. Interestingly, a cluster of metabolites named as cluster C1, of which accumulation was almost exclusively associated to the combination of moderate drought and *T. evansi* infestation, was identified (highlighted in blue, Figure 5). As in clusters C5 and C6, metabolite levels were more intense at one-hpi and decreased afterwards, although these differences were less pronounced than in the metabolites included in *T. evansi*-associated clusters C5 and C6. This group comprised metabolites such as the carbohydrate trehalose, the amino acid L-proline, *N*-*methylanthranilate*, and the phospholipids glycerophosphocholine and phosphatidyl ethanolamine, the first known for their involvement in abiotic stress responses [25,43]. Moreover, two metabolites tentatively annotated as an unknown carboxylic acid and a glycosylated oleoyl tyrosine also accumulated in response to *T. evansi* infestation alone.

## 3. Discussion

### 3.1. Moderate Drought Stress Effects on Tomato Plants

Plant drought stress tolerance is a complex trait, comprising plant physiological and metabolic changes that include fast plant responses such as stomatal closure as well as middle-long term responses such as induction of stress tolerance genes associated to the accumulation of sugars and free amino acids used as plant osmolytes [25]. At the hormonal level, the increase of endogenous ABA levels is the signal controlling plant responses to water stress, the reason why ABA is considered the key player controlling plant adaptation to drought [44,45]. Moderate drought-stress conditions assayed in this work produced a 1.5-fold increase of endogenous ABA levels after seven days, leading to a reduction of 33% in the leaf stomatal conductance with respect to control values together with a ~22-fold induction of the drought-related *RAB18* gene expression. Interestingly, the *AOC* gene expression was increased in plants under moderate drought stress conditions after two days but no modification of JAs levels was observed despite their reported role in water stress responses, suggesting that more severe water stress conditions are required to efficiently trigger jasmonate accumulation [46,47]. Despite differences with other studies potentially related to the tomato cultivars used and the specific stress conditions applied, this and previous water stress studies in tomato have revealed similar trends: an increase of endogenous ABA levels, a reduction of leaf gs, an upregulation of ABA-dependent genes involved in drought response accompanying an accumulation of metabolites with putative osmoprotectant and stress acclimation function [45,46,47,48].

### 3.2. Early Tomato Molecular Responses to T. evansi Attack

Early induction of plant defences in response to generalist phytophagous spider mite *T. urticae* is associated to a general reprograming of plant transcriptome which is mainly controlled by JA and SA, that are accumulated in response to spider mite infestation [15,17,48]. In tomato, this genetic reprograming includes the induction of genes involved in hormone and specialized metabolites biosynthesis such as alkaloids, and the expression of genes coding for proteins involved in plant defences [17,48]. For instance, it has been reported that the expression of *AOC* and *MYC2* genes, involved in JAs biosynthesis and signaling, respectively, are induced in tomato cv Castelmart plants as early as three hours after *T. urticae* infestation [16]. On the other hand, it has been described that the solanaceous crops specialist spider mite *T. evansi* supresses the JA production and JA-dependent tomato anti-mite defenses [22,49], producing the gene repression of JA-dependent *MYC2* and protein inhibitors *PI-Ia* and *PI-IIc* genes at two-, four-, and/or seven-days post infestation after a 24 h post infestation transient induction [21,22,33,49]. Similarly, in this work, we found no significant alteration of JA levels and associated gene expression in mite infested plants despite an early transient induction at one-hpi, pointing at a mechanism of *T. evansi*-mediated suppression of JA biosynthesis identical to that described previously [22,50]. Moreover, we observed that SA levels increased in *T. evansi* infested plants after three- and eight-hpi, and it is feasible that this accumulation continues at 24 h of infestation as Alba et al. [22] observed with two *T. evansi* populations, confirming the relevant role of the SA hormone in the initial steps of tomato defenses suppression by putative promoting the JA-SA antagonism [22,23,49,50].

Plant metabolite profiling showed significant metabolic changes in tomato red spider mite infested plants compared with control plants and between infestation time points, indicating that at the metabolic level, the response of tomato plants to *T. evansi* infestation is a very dynamic process with a clear temporal component. We observed faster changes in the plant amino acid and carbohydrate metabolism, that together with the genetic suppression of the molecular tomato defense responses, may act as a part of the colonization strategy of the tomato red spider mite. It is known that tomato red spider mites produce protein effectors to suppress plant defenses as part of their colonization strategy and it is reasonable to speculate that they are responsible for these faster metabolic changes [23,50]. Hence, our data indicated that the molecular processes promoted by these effectors were started as early as one hour after mite infestation, inducing a high accumulation of certain metabolites that was reduced afterwards. Interestingly, metabolites induced by *T. evansi* attack also responded to combined stress condition but showed significantly lower levels, suggesting that, somehow the presence of drought downregulates the mechanisms that trigger their accumulation.

### 3.3. Early Tomato Molecular Responses to T. evansi Attack in Combination with Moderate Drought Stress

In tomato, it has been described that the combination of moderate drought stress and spider mite infestation resulted in an increase of tomato leaf damage, indicating the existence of a synergistic effect that increases the spider mite performance and population growth [32,33]. In the present work, the early response of tomato to the combination of moderate drought and tomato red spider mite combination has been analyzed. Hormonal profiling of tomato under combined stress showed that JA and ABA levels increased with a similar trend in plants subjected to moderate drought alone or in combination with *T. evansi* infestation. However, SA levels were different between *T. evansi* infested plants alone or in combination with moderate drought stress at three-hpi, indicating that the increased mite performance under moderate drought may be due to the early disturbance of SA levels. The gene expression pattern of the SA-dependent *PR-1a* also suggests that the SA-signaling pathway is disturbed at three-hpi, but more SA-dependent genes must be analyzed to support this conclusion. On the other hand, the gene transcription analysis also confirmed the disturbance of tomato drought-dependent responses. The expression pattern of some drought-induced genes changed in dual-stressed plants, with a reduction of *DREB2*, *RAB18*, *AOC*, and *JA2* gene expression at eight-hpi compared with the expression in the moderate drought-stressed plants, indicating that spider mites could modify drought-dependent tomato responses, in a way facilitating its colonization and proliferation. However, in order to confirm and quantify the modification of drought-dependent response by *T. evansi* attack, a wide expression analysis as RNAseq must be performed.

We have shown that tomato plants exhibit an early modulation of their metabolism in response to drought and tomato red spider mite combination that leads to a metabolic state with similar metabolic variability than the metabolic state of drought-stressed samples. Interestingly, *T. evansi* infested plants showed metabolic changes during the infestation time that also lead to a metabolic state with a variability respect to control plants similar to that of moderate drought-stressed and dual-stressed samples. For instance, the concurring action of drought and *T. evansi* infestation induced the accumulation of proline and the carbohydrate trehalose which have been associated with stress protection but also as putative signaling metabolites in plant-herbivory interactions [21,51]. Interestingly, this accumulation was highest at one-hpi and reduced thereafter, suggesting that *T. evansi* infestation is the main factor driving its accumulation although drought stress seems to be a requisite for it to take place. Although the actual role of both metabolites cannot be ascertained with the current experimental design, it seems clear that their accumulation is further exacerbated by the simultaneous imposition of drought and spider mite infestation which is in agreement with the idea that the increment of mite performance is a reflect of significant changes in the nutritional quality of tomato plants [33]. It has been described the JA-dependent reduction of leaf carbohydrate accumulation in *Nicotiana attenuata* as a metabolic response aimed at reducing growth *Manduca sexta* caterpillars, which reinforce the idea that *T. evansi* effectors not only suppress plant defenses response but also boost up metabolic changes to increment mite performance [52,53]. Moreover, there were exclusive responses to stress combination that were not shared with individual moderate drought or *T. evansi* infestation treatments. To this respect, moderate drought did not induce any clear specific metabolic response, but its presence clearly modulated the metabolic response of dual-stressed tomato plants. Moreover, *T. evansi* infested samples did show a number of metabolites that were associated to this factor but also to the stress combination, although their levels were much lower, suggesting that the mechanism that triggers the exclusive accumulation of certain metabolites in dual-stressed samples could, at the same time, downregulate metabolites that are highly accumulated in tomato plants subjected to *T. evansi* infestation alone.

The role of ABA in plant biotic interactions and its crosstalk with SA and JA have been extensively described to fine-tune the outcomes between growth and defence, allocating different roles for ABA based on the pathogen/pest and the plant species, and unfortunately for plants, some pathogens have learned how to used it to promote their growth [28,54]. For instance, the pathogenic bacteria *Pseudomonas syringae* pv *tomato* (*Pst*) DC3000 not only produces coronatine to promote JA-dependent responses, but also increases plant ABA levels to antagonize SA-dependent defences [54] through the induction of the ABA-dependent degradation of the SA receptor NPR1 via the CUL3^NPR3/NPR4^ complex-mediated 26S proteasome pathway [55], among others mechanisms. However, the green peach aphid *Myzus persicae* induces the production of ABA in Arabidopsis which reduces the production of anti-digestive compounds called glucosinolates, suggesting a direct function of ABA in the control of plant defences against this aphid [56]. More evidence is needed to know if the increased performance of the tomato red spider mite under moderate stress conditions is caused by ABA regulating some plant defence responses to spider mites or if ABA is modulating plant responses to tomato red spider mite infestation via crosstalk with the SA biosynthesis or signaling pathways.

## 4. Materials and Methods

### 4.1. Plant Material and Mite Rearing

Commercial *Solanum lycopersicum* cv Moneymaker (MM) plants were grown from seeds in 40-well trays until they show three expanded leaves and then were transferred to 2.5 L pots (diameter: 16 cm, height: 15 cm) (Maceflor©, Valencia, Spain) filled with 600 g of Universal growing medium “Compo sanaR” (Compo GmbH, Münster, Germany). A colony of *T. evansi* derived from the Nice strain collected in Beausoleil (South of France) was provided by Dr. Maria Navajas (CBGP, Montpellier, France). Mites were maintained on detached MM tomato leaves placed on ventilated plastic cages as previously [21]. Plants and mite cages were maintained in climate rooms at 25 ± 1 °C and a 16 h light/8 h dark photoperiod.

### 4.2. Drought Stress Regime

Drought stress was applied by withholding irrigation as described by Ximénez-Embún et al., [32]. In brief, tomato plants were well-watered until they developed four to five fully expanded leaves, then non-stress and moderate drought stress regimes were imposed. Control plants were watered regularly to maintain the percentage of saturation weight (SW) between 100 to 78%. For moderate drought stress conditions, watering was stopped for seven days until they arrive at SW between 45 to 55%. In both experiments, moderate water-stressed plants were over the wilting point associated with severe drought stress that was previously established in our experimental conditions [32]. Score of plant physiological parameters under drought stress was done on one of the sub-terminal leaflet of the fourth leaf. The plant physiological parameters recorded were (a) stomatal conductance (gs) using a leaf porometer (SC-1 Decagon-T, Pullman, WA, USA), (b) variations in maximum quantum yield of photosystem II photochemistry (Fv/Fm), using a FluorPen FP 100 (PSI, Drasov, Czech Republic), and (c) plant growth that was estimated by measuring the stem length (distance between the soil and the terminal bud). Plant material was sampled three hours after lights of the climate room turned on at the indicated day. Two experiments were performed with seven biological replicates per treatment, three in the experiment 1 and four in the experiment 2.

### 4.3. Tomato-Spider Mite Bioassays

Experiments were carried out in a climate room showing relative humidity of 50 ± 5% at 25 °C ± 1 °C and a 16 h light/8 h dark photoperiod. Tomato plants were assigned to four different groups combining two treatments: uninfested well-watered plants (Control); uninfested drought-stressed plants (Drought); infested well-watered plants (*T. evansi*); and infested drought-stressed plants (Drought + *T. evansi*).

Seven days after stopping irrigation plants were infested with 100, *T. evansi* females of random age which were collected from the laboratory colony by using a vacuum pump D-95 (Dinko S.A., Barcelona, Spain) with a sucking power of 10–50 mmHg, connected to a modified polypropylene microtube. They were placed on one of the sub-terminal leaflets of the four leaf. All plants (infested and non-infested) were set up in the climate room following a complete randomized block design. Plant material was collected, ground in liquid nitrogen to a fine powder, and stored at −80 °C until analysis. Plant material was sampled at the indicated time. Samples of the water stress imposition were harvested three hours after lights in the climate room were turned on and samples of the time course where harvested starting at one-hour time point, which correspond to three hours after lights were turned on. Two experiments were performed with seven biological replicates per treatment, three in the experiment one and four in the experiment two.

### 4.4. Plant Hormone and Metabolite Profiling

Analysis of plant hormones (ABA, SA, and JA) was attained with LC/ESI-MS/MS (Waters Acquity SDS UPLC coupled to a TQ-D, Micromass Ltd., Manchester, UK) on aqueous plant extracts (c.a. 10 mg dry weight each replicate) as in Ximenez-Embun et al., (2018). Hormone quantitation was performed with Masslynx v. 4.0 software (Micromass Ltd., Manchester, UK) after external calibration with standard samples containing known amounts of each plant hormone.

Non-targeted metabolite profiling was performed using hydrophilic interaction liquid chromatography (HILIC) coupled to hybrid quadrupole-time of flight mass spectrometry (QTOF-MS) essentially as described previously [57] with some modifications. HILIC separation was performed on a 2.1 × 100 mm ACQUITY UPLC 1.7 µm BEH amide column (Waters Corp. Ltd., Milford, MA, US) using acetonitrile:water, 95:5 (v/v) (solvent A) and acetonitrile:water, 2:98 (v/v) (solvent B), both supplemented with ammonium formate at 0.063% and 0.126%, respectively, as solvents at a flow rate of 300 µL min^−1^. During chromatographic runs, column temperature was maintained at 40 °C. All solvents and mobile phase modifiers were of LC/MS grade.

Tomato leaf samples (~150 mg fresh weight) were extracted by ultrasonication in 300 µL of 80% aqueous methanol supplemented with ribitol (5 mg L^−1^) as internal standard for relative quantitation. After extraction, samples were centrifuged at 10,000 rpm for 10 min at 4 °C and the supernatants recovered. Subsequently, supernatants were diluted 1:1 with acetonitrile (LC/MS grade) and filtered through 0.2 µm PTFE syringe filters directly into chromatography vials.

Mass chromatographic data were acquired with a hybrid quadrupole/time-of-flight mass spectrometer equipped with an electrospray source that was operated in positive and negative ionization modes within the 40–800 amu mass range. Nitrogen was used both as nebulization and desolvation gas (60 and 800 L h^−1^ and 350 °C temperature, respectively). During measurements, capillary and cone voltages were set at 3.5 kV and 30 V for positive electrospray and 2.3 kV and 30 V for negative electrospray, respectively. An additional acquisition function to obtain collision-induced dissociation (CID) information was set by performing a voltage ramp between 6–40 eV. To ensure accurate mass data acquisition, a lockmass reference (leucine-enkephalin [M + H]^+^ 556.2771 and [M − H]^−^ 554.2514) was regularly infused during runs. Data from each ionization mode were processed independently.

Mass chromatographic data were extracted and processed with XCMS [58] and peaks annotated with CAMERA [59]. Peak areas were normalized to internal standard area and actual sample weight before statistical analyses. Differences between control and treated plants were assessed by means of students’ *t*-test. Significantly-altered mass chromatographic features were subsequently identified as individual compounds by matching mz and retention time values with those of authentic standards or tentatively annotated by matching experimental mass spectra in public databases (metlin, Massbank or HMDB).

For dendrogram and heat map calculation, distance metrics of the normalized peak areas were Euclidean distances and a complete linkage clustering method was used.

### 4.5. Quantification of Gene Expression via qRT-PCR

The expression levels of several hormone response-genes were measured by qRT-PCR according to Ximénez-Embún et al. [21]. qRT-PCR was carried out in a Corbett Rotor Gene 6000 real-time cycler (Qiagen) using the Brilliant III Ultra-Fast SYBR Green QPCR Master Mix (Agilent Technologies, Santa Clara, CA, USA). Raw gene expression data were efficiency-corrected and gene expression was transformed to normalized relative quantities (NRQ) by using multiple house-keeping reference genes which allow the sample internal normalization [60]. The NRQ values for each time point were later normalized with respect to the expression of its control expression value to reduce the effect of the experiment replica. The primers used are listed in Table S5.

### 4.6. Statistical Analysis

All data were checked for the assumptions of normality and heteroscedasticity, and transformed if necessary. Stomatal conductance, phytohormone and metabolite data were log10(x) transformed and gene-expression data (NRQ values) were finally expressed as expression ratio relative to the expression in control plants. Different types of statistical analysis were performed depending on the design and purpose of each experiment: (1) a two-tailed Student’s *t*-test was performed to determine the effects of moderate drought imposition in the gene expression and (2) a two-way ANOVA with the plant treatment and the experiment replica as fixed factors (except for gene expression data) was performed to analyze plant physiological parameters, phytohormone levels, gene expression, and metabolite profiling. When the factor was significant, Tukey HSD post-hoc test was performed to compare treatments. For the statistical analysis, the IBM SPSS Statistics 26.0 software (Chicago, IL, USA) was used.

## Supplementary Materials

The following are available online at https://www.mdpi.com/2223-7747/9/9/1131/s1, Figure S1: Tomato characterization during moderate drought imposition. Saturation weight measurements in moderate drought tomato experiment. Stem growth and photosynthetic efficiency at two, five, and seven days after water withdrawal. Figure S2: Hormone profiling of tomato plants at two, five, and seven days after water withdrawal. Figure S3: Stress-marker gene expression analysis of tomato plants at two, five, and seven days after water withdrawal. Table S1: Statistical detailed results of the Student’s *t*-test performed at two, five, and seven days after water withdrawal for hormone profiling and gene expression analysis. Table S2: Statistical detailed results of the two-way ANOVA and post-hoc performed during drought, *T. evansi* infestation and their combination at one-, three-, and eight-hours post infestation for hormone profiling and gene expression analysis. Table S3: Detailed results of the tomato metabolite profiling at two five and seven days after water withdrawal. Table. S4: Detailed results of the tomato metabolite profiling under drought, *T. evansi* infestation and their combination at 1, three and eight hours post infestation. Table S5: Detailed information of the qPCR primers used in this study.
