# Supplementary material for: Early Molecular Responses of Tomato to Combined Moderate Water Stress and Tomato Red Spider Mite Tetranychus evansi Attack
Source: Plants (Basel). 2020 Aug 31;9(9):1131. doi: 10.3390/plants9091131 (PMC7570366; doi:10.3390/plants9091131)
Supplement: Supplementary file 1 [file plants-09-01131-s001.pdf]

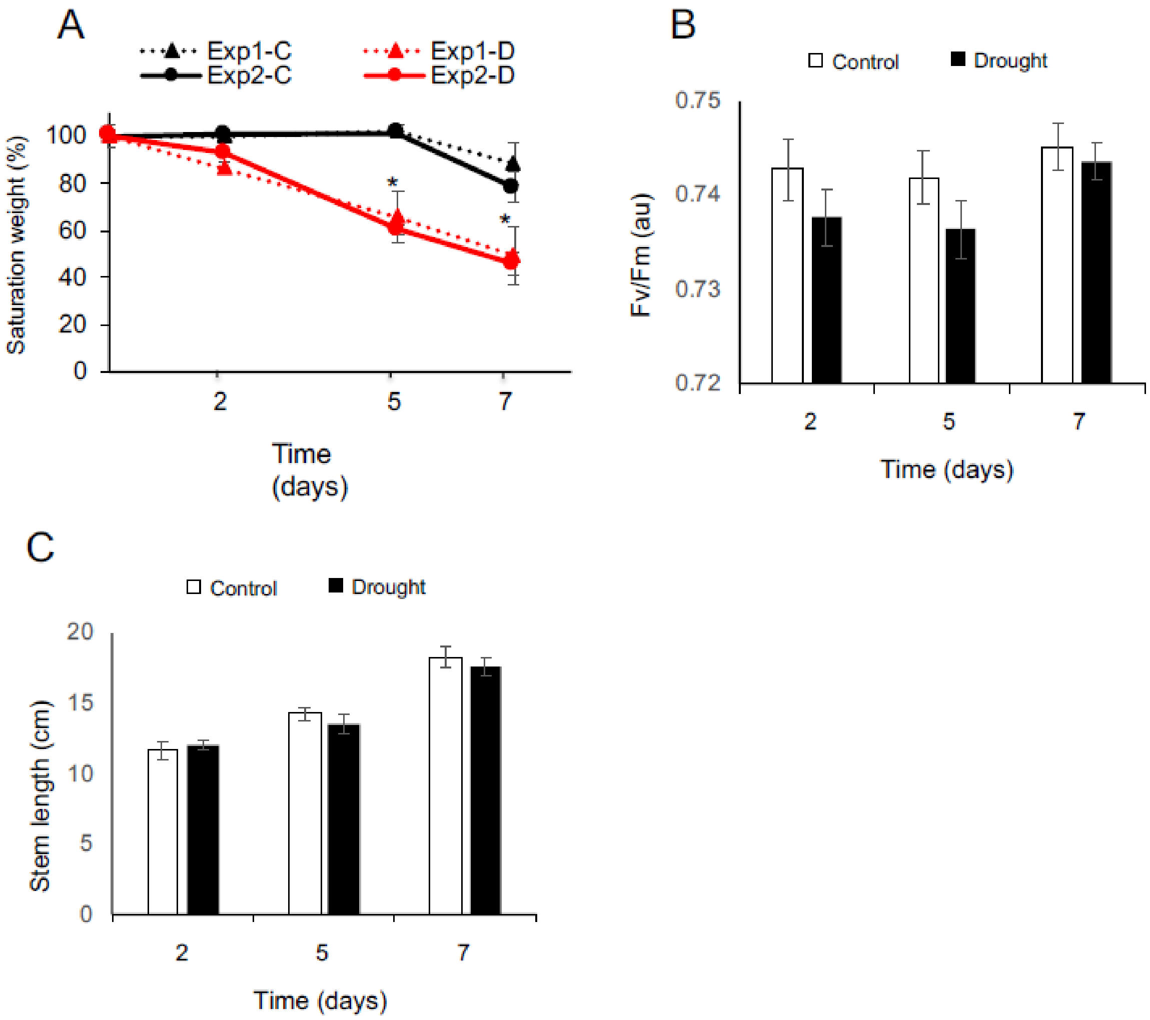

Figure S1. Tomato characterization during moderate drought imposition. (**A**) saturation weight measurements in moderate drought tomato experiment 1 (Exp1, dotted lines) and experiment 2 (Exp2, continuous lines) during the water stress imposition in control plants (black line) and drought stressed plants (red line), (**B**) Stem growth (stem length) and (**C**) photosynthetic activity (Fv/Fm) in drought stressed plants after two, five and seven days of water withdrawal. Data are mean ± SE of seven replicates/treatment from two independent experiments. Asterisk indicates significant differences of each treatment respect its control (Student's t-test, $p < 0.05$).

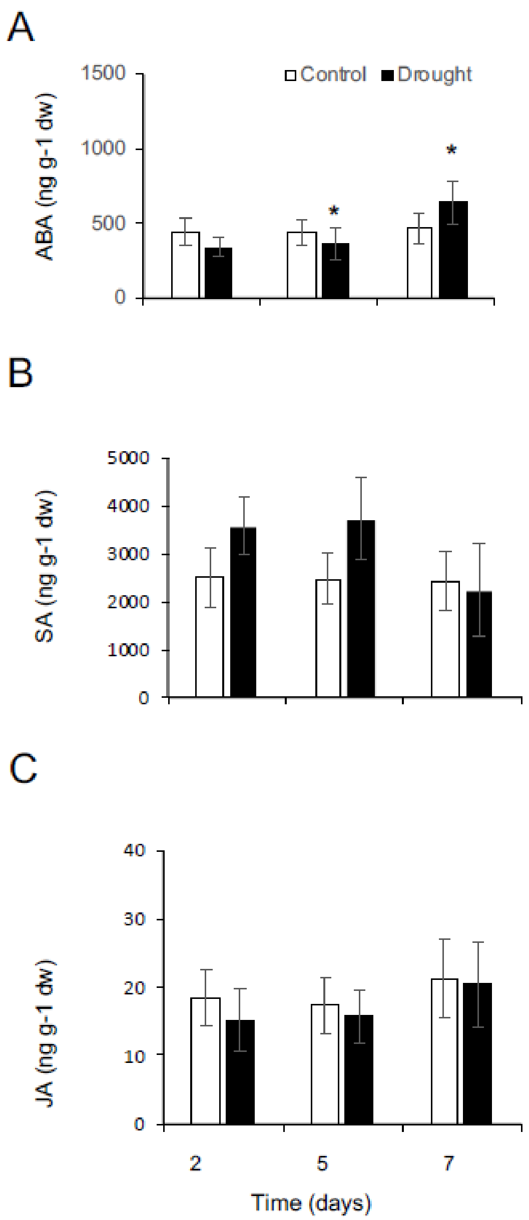

Figure S2. Hormone profiling of tomato plants under moderate water stress conditions. (**A**) ABA, (**B**) SA and, (**C**) JA endogenous levels in well-watered plants (Control) and drought-stressed plants (Drought) at two, five and seven days after water withdrawal. Data are mean ± SE of seven replicates/treatment from two independent experiments. Plant treatment was analysed by two-way ANOVA considering the experiment replica as a fixed factor. Asterisk indicates significant differences of each treatment respect its control ($p < 0.05$).

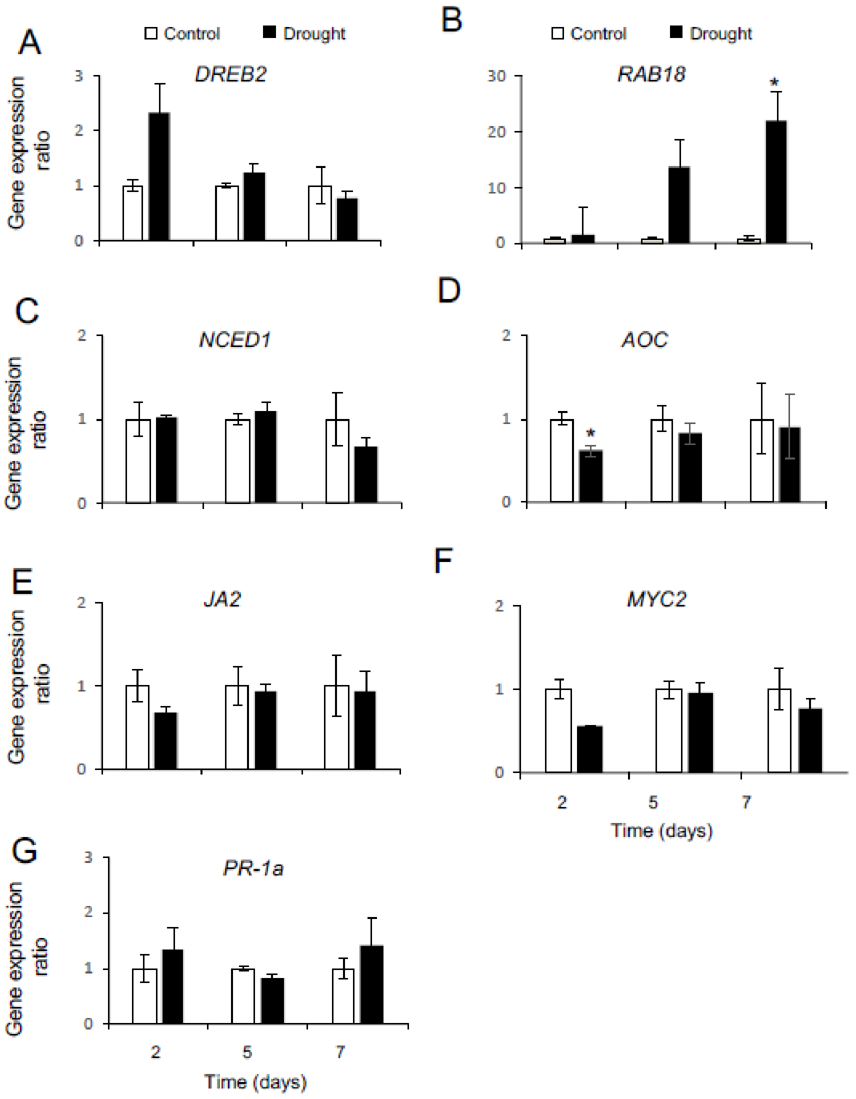

Figure S3. Stress-marker gene expression analysis of tomato plants under moderate drought stress. The expression of (**A**) DREB2, (**B**) RAB18, (**C**) NCED1, (**D**) AOC, (**E**) MYC2, (**F**) JA2, and (**G**) PR-1a genes were analysed in well-watered plants (Control) and drought-stressed plants (Drought) at two, five and seven days after water withdrawal. Gene expression calculated as normalized relative quantities (NRQ) values are represented as gene expression ratio compared to its control sample for each time point. Data are mean ± SE of seven replicates/treatment from two independent experiments. Plant treatment was analysed by two-tailed Student's t-test. Asterisk indicates significant differences of each treatment respect its control ($p < 0.05$).

Table S1. Detailed results of the statistical analysis for phytohormones and gene expression during water stress imposition. **A**) ANOVA analysis (drought and replica as fixed factors) for phytohormones data. Data were log10(x) transformed. In bold the significant factors ($p < 0.05$). **B**) Student's t-test analysis for gene expression data. Data were normalized with respect to their corresponding control within each replica. In bold the significant factors ($p < 0.05$, adjusted for not equal variances). Degrees of freedom —df, p value—*p*, t-Student statistic value —t and F-statistic value—F are indicated.

A)

		**Drought**			**Replica**			**Drought*Replica**		
		**F**	**df**	***p***	**F**	**df**	***p***	**F**	**df**	***p***
**ABA**	2d	2.926	1,10	0.118	91.553	1,10	**0.000**	4.145	1,10	0.069
	5d	8.023	1,10	**0.018**	216.718	1,10	**0.000**	0.129	1,10	0.727
	7d	20.081	1,10	**0.001**	267.959	1,10	**0.000**	0.789	1,10	0.395

**SA**	2d	3.752	1,10	0.081	15.937	1,10	**0.003**	0.086	1,10	0.775
	5d	4.138	1,10	0.069	19.094	1,10	**0.001**	0.428	1,10	0.528
	7d	1.087	1,10	0.322	11.963	1,10	**0.006**	0.023	1,10	0.882
**JA**	2d	3.563	1,10	0.088	28.411	1,10	**0.000**	0.064	1,10	0.806
	5d	1.135	1,10	0.312	77.164	1,10	**0.000**	0.380	1,10	0.552
	7d	0.026	1,10	0.876	43.181	1,10	**0.000**	0.096	1,10	0.764

B)

	2 days			5 days			7 days		
	t	df	*p*	t	df	*p*	t	df	*p*
				**Genes**					
**DREB2**	−0.964	6	0.388	−1.706	6	0.163	0.969	6	0.402
**RAB18**	−3.111	6	0.053	−2.108	6	0.124	−4.211	6	**0.024**
**NCED1**	−1.186	6	0.317	−0.371	6	0.733	0.541	6	0.626
**AOC**	3.256	6	**0.044**	−0.152	6	0.889	−0.924	6	0.423
**MYC2**	2.760	6	0.063	0.503	6	0.646	1.298	6	0.283
**JA2**	2.990	6	0.054	−0.286	6	0.793	−0.758	6	0.504
**PR1a**	1.211	6	0.306	−0.391	6	0.722	−0.001	6	0.999

Table S2. Detailed results of the statistical ANOVA analysis for phytohormones and gene expression during the stress combination. **A**) ANOVA analysis for phytohormones. Drought and *T. evansi* treatments, and the experiment replica were considered as fixed factors. Data were log10(x) transformed. All factors' interactions were studied but only Drought**T. evansi* interaction is shown. **B**) ANOVA analysis for gene expression. Drought and *T. evansi* treatment were considered as fixed factors. Data were normalized with respect to their corresponding control within each replica. In bold the significant factors ($p < 0.05$). Degrees of freedom —df, p value—*p*, and F-statistic value—F are indicated.

A)

		Drought			*T. evansi*			Drought**T. evansi*			Replica		
		F	df	*p*	F	df	*p*	F	df	*p*	F	df	*p*
**ABA**	**1h**	34.800	1,20	**0.000**	2.167	1,20	0.157	1.704	1,20	0.207	378.242	1,20	**0.000**
	**3h**	14.946	1,20	**0.001**	0.282	1,20	0.601	0.514	1,20	0.482	114.391	1,20	**0.000**
	**8h**	2.410	1,20	0.136	0.593	1,20	0.450	0.257	1,20	0.618	73.256	1,20	**0.000**
**SA**	**1h**	0.389	1,20	0.540	0.084	1,20	0.774	1.039	1,20	0.320	15.494	1,20	**0.001**
	**3h**	0.527	1,20	0.476	22.212	1,20	**0.000**	15.585	1,20	**0.001**	1.238	1,20	0.279
	**8h**	0.081	1,20	0.779	21.575	1,20	**0.000**	0.000	1,20	0.983	13.586	1,20	**0.001**
**JA**	**1h**	1.077	1,20	0.312	8.912	1,20	**0.007**	0.642	1,20	0.432	89.280	1,20	**0.000**
	**3h**	0.411	1,20	0.529	3.441	1,20	0.078	2.081	1,20	0.165	304.401	1,20	**0.000**
	**8h**	0.124	1,20	0.728	1.177	1,20	0.291	0.794	1,20	0.384	119.745	1,20	**0.000**

B)

		Drought			*T.evansi*			Drought**T.evansi*		
		F	df	*p*	F	df	*p*	F	df	*p*
**DREB2**	**1h**	0.389	1,12	0.544	2.361	1,12	0.150	0.440	1,12	0.520
	**3h**	0.513	1,12	0.487	0.158	1,12	0.698	0.541	1,12	0.476
	**8h**	13.610	1,12	**0.003**	0.638	1,12	0.440	14.068	1,12	**0.003**
**RAB18**	**1h**	21.483	1,12	**0.001**	0.756	1,12	0.401	0.030	1,12	0.865
	**3h**	41.789	1,12	**0.000**	8.802	1,12	**0.012**	3.072	1,12	0.105
	**8h**	68.623	1,12	**0.000**	41.531	1,12	**0.000**	0.531	1,12	0.480
**NCED1**	**1h**	0.149	1,12	0.706	0.147	1,12	0.708	0.005	1,12	0.947
	**3h**	5.296	1,12	**0.040**	0.347	1,12	0.567	0.235	1,12	0.636
	**8h**	0.191	1,12	0.670	1.578	1,12	0.233	0.811	1,12	0.386
**AOC**	**1h**	0.639	1,12	0.439	0.180	1,12	0.679	0.119	1,12	0.736
	**3h**	4.285	1,12	0.061	0.077	1,12	0.786	0.180	1,12	0.679
	**8h**	28.501	1,12	**0.000**	3.172	1,12	0.100	1.742	1,12	0.212
**MYC2**	**1h**	0.036	1,12	0.853	0.001	1,12	0.978	0.832	1,12	0.380
	**3h**	1.479	1,12	0.247	4.086	1,12	0.066	3.304	1,12	0.094
	**8h**	0.210	1,12	0.655	0.646	1,12	0.437	1.492	1,12	0.245
**JA2**	**1h**	0.635	1,12	0.441	0.343	1,12	0.569	0.154	1,12	0.702
	**3h**	4.069	1,12	0.067	0.045	1,12	0.835	0.198	1,12	0.664
	**8h**	32.578	1,12	**0.000**	3.277	1,12	0.095	1.879	1,12	0.196
**PR1a**	**1h**	0.001	1,12	0.976	0.012	1,12	0.913	0.001	1,12	0.974

**3h**	0.012	1,12	0.913	6.424	1,12	**0.026**	277.366	1,12	**0.000**
**8h**	1.395	1,12	0.260	0.725	1,12	0.411	0.713	1,12	0.415

Table S3. Annotation of mass chromatographic features in initial drought imposition (annotation level: 1, matched against authentic standard; 2, putative annotation by matching mass spectra with public libraries; 3, tentative annotation by partial matching of mass spectra with public libraries). Quantified ions are highlighted in bold. nd, not determined. Mass to charge ratio—mz and retention time in seconds—rt, are indicated.

Annotation	*mz*	rt [s]	ion type	Annotation level
**oleoylcarnitine glucoside**	**588.390142**	**965.066**	**[M + H] + 587.382**	2
	426.333544	965.066	[M + H − C6H10O5] + 587.382	
	86.0564556	976.708	[M + H − H20] + 103.064	
	104.071781	978.09	[M + H] + 103.064	
**Benzyl O-[arabinofuranosyl-(1->6)-glucoside]**	**403.16447**	**578.9025**	**[M + H] + 402.156**	2
	91.0554303	578.488		
	109.029845	578.482		
	115.040556	578.481		
	133.058687	578.48		
	253.110004	578.487		
	295.104284	578.481		
	420.187394	578.488		
	425.145315	578.488		
	441.117763	578.481		
**Choline**	**104.108154**	**482.6835**	nd	2
	97.0297526	578.488		
**Phosphatidyl choline phospholipid #1**	**696.545366**	**59.609**	**[M + H − H20] + 713.548**	3
	184.07	59.609		
	335.269	59.609		
**Phosphatidyl choline phospholipid #2**	**696.545771**	**174.861**	**[M + H − H20] + 713.548**	3
	351.254658	175.9	[M + H − C6H10O5] + 512.298	
	262.2793	174.6		
**Phosphatidyl Serine phospholipid**	**726.453421**	**87.709**	**[M + H] + 725.445**	3
**Linalool 3,7-oxide beta-primeveroside**	**465.258235**	**517.361**	**[M + H] +**	2
	311.259127			
	447.250874			

**L-lyxo-phytosphingosine #1**	**318.301664**	**91.963**	**[M + H] + 317.294**	**2**
	274.275582	91.958	[M + H − C4H8] + 329.331	
	123.404008	90.886	[M + H] + 122.396	
**octanal**	**274.275564**	**190.843**	nd	
**gingerol**	**323.222828**	**194.307**	**[M + H] +**	**2**
**fatty acid**	**275.202357**	**109.184**	**[M + H] + 274.196**	**3**
**Glutamine**	**147.077667**	**1011.27**	**[M + H] +**	
	130.067	1011.27	[M − NH3] +	
	84.064	1011.27		
**Diacylglycerol**	**591.498776**	**408.5985**	**[M + H] + 590.493**	**3**
	573.433		[M − H2O] +	
	313.28			
**Betaine**	**118.087852**	**698.208**	nd	**2**
**feruloyl glucose**	**374.145347**	**648.644**	**[M + H + NH3] + 356.115**	**2**
	379.106336	648.022	[M + Na] + 356.115	
	193.14			
**Isopentyl gentiobioside**	**430.229259**	**597.936**	**[M + H + NH3] + 412.193**	**2**
	435.183199	598.984	[M + Na] + 412.193	
	451.154387	597.935	[M + K] + 412.193	
**nicotinate beta-D-ribonucleotide**	**336.063185**	**537.843**	**[M + H] + 335.054**	**2**
**lucuminic acid**	**464.176084**	**498.969**	**[M + H + NH3] + 446.142**	**2**
	485.105438	499.997	[M + K] + 446.142	
	153.056181	499.986		
**(S)-5'-Deoxy-5'-(methylsulfinyl)adenosine**	**314.093684**	**518.394**	nd	**2**
	136.065949	518.396	adenine moiety	
**kaempferol hexoside deoxyhexoside**	**595.168464**	**592.721**	nd	**1**
	287.057074	592.722		
	449.109487	592.722		
**L-lyxo-phytosphingosine #2**	**318.299626**	**186.68**	nd	**2**
	140.081696	187.196		
**ornithine moiety**	**133.101825**	**142.501**	nd	**3**
**alkaloid**	**476.285**	**622.375**	nd	**3**
**guanine**	**152.059342**	**584.042**	nd	**1**

disaccharide	365.106551	1046.22	[M + Na] + 342.118	2
	162.07776	1051.34		
polymethoxylated flavone	361.091262	661.5015	[M + H] + 360.085	3
maltose	409.095047	953.295	[M – H + NaCOOH] – 342.117	2
pimpinellin	245.043503	977.829	[M – H] – 246.051	2
2-Dodecylbenzenesulfonic acid	325.18514	73.3335	[M – H] – 326.192	2
	68.99741			
	112.985972			
	297.152415			
	311.169795			
pyroglutamic	128.036542	1006.08	nd	1
benzamide	120.046797	166.376	[M – H] – 121.054	2
cyanidin glucoside	447.093937	358.009	nd	1
O-alpha-Glucopyranosyl-(1-4)-O-alpha-xylopyranosyl-(1-4)-O-alpha-xylopyranosyl-(1-4)-glucopyranose	605.1933	1338.74	nd	2
purine	119.036041	812.2705	nd	3
synapoyl glucose	385.114155	206.226	[M – H] – 386.122	2
	205.0514	206.239	[M – H – H20] – 224.071	
(2S)-2-Butanol O-[b-D-Apiofuranosyl-(1->6)-b-D-glucopyranoside	367.159858	600.102	[M – H] – 368.167	2
	235.117691	599.082	[M – H – C5H8O4] – 368.167	
7-Methoxy-2-methylisoflavone	265.09483	880.007	nd	2
Malondialdehyde	71.0161559	704.6405	nd	2
Inositol cyclic phosphate	241.014144	938.361	nd	2
Unknown pentoside	229.10901	631.376	[M – H – C5H8O4] – 362.159	3, nd
Uridine diphosphate-N-acetylglucosamine	606.075026	1294.19	[M – H] – 607.082	2
O-Caffeoylquinic acid #1	353.088102	835.9085	nd	2
O-Caffeoylquinic acid #2	353.087878	916.816	nd	2
Succinic acid semialdehyde	101.025621	817.838	nd	2
	73.048	833.4		
peroxynitrite/nitrate	61.9902408	148.9055	nd	3

Table S4. Annotation of significantly-altered mass chromatographic features in the experiments involving concurring spider mite infestation and drought (annotation level: 1, matched against authentic standard; 2, putative annotation by matching mass spectra with public libraries; 3, tentative annotation by partial matching of mass spectra with public libraries). Quantified ions are highlighted in bold. nd, not determined. Mass to charge ratio —mz and retention time in seconds—rt, are indicated.

Annotation	*mz*	rt [s]	ion type	Annotation level
**Glycerophosphocholine**	**258.111749**	**1028.59**	**[M + H] + 257.104**	**2**
	104.10798	1028.585		
	184.074999	1028.585		
**Phosphatidyl ethanolamine (PE)**	**770.59156**	**79.019**	**[M + H] + 769.579**	**3**
	335.25977	81.101		
	563.46906	79.0395	[M + H − C2H4] + 590.495	
	567.439617	80.0615	[M + Na] + 544.451	
	585.45095	81.109		
	600.440986	80.954	[M + H − C2H4] + 627.466	
	613.482474	81.0035	[M + Na] + 590.495	
	628.475041	80.961	[M + H] + 627.466	
	742.54983	79.0305	[M + H − C2H4] + 769.579	
	762.487567	81.112		
	764.533071	79.9185		
**Anthranilic acid**	**138.056529**	**751.491**	**[M + H] + 137.05**	**1**
	92.0505177	751.91	[M + H − HCOOH] + 137.05	
	94.0663011	751.6895	[M + H − CO2] + 137.05	
**Diterpenoid glycoside**	**515.321924**	**155.484**	**[M + H] + 514.318**	**3**
	261.222667	155.4895	[M + H − C2H4] + 288.254	
	335.259657	155.4895	[M − H2O] + 352.2694	
	353.269484	155.4895	[M + H − C6H10O5] + 514.318	
**Linoleoyl glycerophosphocholine**	**520.340134**	**530.146**	**[M + H] + 519.333**	**2**
	502.324566	530.1465	[M + H − H20] + 519.333	
**Octylamine #1**	**130.160162**	**66.5**	nd	**3**
**L-proline**	**116.072152**	**797.242**	**[M + H] +**	**1**
	70.0664826	797.538	[M + H − HCOOH] + 69.0593	
**Phosphatidylserine (PS)**	**554.344171**	**573.223**		**2**
	534.319881	572.1815		
**Glucocerebroside-like**	**714.552105**	**173.863**	**[M + H] + 713.546**	**3**
	552.500286	173.863	[M + H − C6H10O5] + 713.546	
	696.546507	174.238	[M + H − H20] + 713.546	
**Glycosylated steroid alkaloid tentative**	**594.403809**	**958.885**	**[M + H] + 593.397**	**3**
	295.104998	958.204	[M + H − C6H10O5] + 456.15	
	428.351826	960.2655	[M + H] + 427.343	
	432.349114	959.52	[M + H − C6H10O5] + 593.397	
**Octylamine #2**	**130.160446**	**197.692**	nd	**3**
**γ-Glutamyl-β-aminopropiononitrile**	**217.131869**	**979.5795**	**[M + H+NH3] + 199.095**	**2**
	158.08417	979.5795	[M + H − COCH2] + 199.095	
	175.116193	979.1165		
**Phosphatidyl glycerol (PG)**	**743.47648**	**94.7655**	**[M + H] + 742.469**	**2**
	489.356499	95.177		
	563.408467	94.7665	[M + H − H20] + 580.415	
	581.421819	94.7655	[M + H − C6H10O5] + 742.469	
**Diacylglycerol #1**	**609.454387**	**90.8165**	**[M + H] + 608.444**	**2**
	123.056443	90.8435	[M + K] + 84.0949	
	191.144259	90.97		
	318.301504	91.8775		
	495.404728	90.815	[M + H] + 494.396	
	517.38243	91.8615	[M + Na] + 494.396	
	597.415776	90.8385	[M + H − C4H8] + 652.48	
**Unknown guanosine derivative**	**312.130152**	**442.952**	**[M + H] + 311.123**	**3**
	180.089289	441.906	[M + H − C5H8O4] + 311.123	
**Glycosyl oleosyl tyrosine tentative**	**608.380073**	**1066.395**	**[M + H] + 607.373**	**3**
	446.327061	1066.385	[M + H − C6H10O5] + 607.373	
**L-Glutamate**	**148.062283**	**1144.895**	**[M + H]+ 147.054**	**1**
	102.056211	1144.9	[M + H − HCOOH] + 147.054	

	130.05145	1143.875	[M + H − H20] + 147.054	
**Trp/anthranilate-derived alkaloid #1**	**160.077523**	**525.4205**	nd	**3**
**Adenine**	**136.065707**	**260.995**	nd	**1**
**Diacylglycerol #2**	**591.498873**	**407.5545**	**[M + H] + 590.493**	**3**
	97.1001582	407.504		
	102.890466	406.16		
	107.381363	405.093		
**Unknown oxylipin**	**211.170449**	**623.9395**	**[M + H] + 210.165**	**3**
	174.142194	623.9545		
	193.160743	622.9025	[M + H − H20] + 210.165	
**Phosphocholine**	**184.075279**	**532.2415**	nd	**2**
**Kaempferol**	**287.057627**	**348.149**	nd	**1, 3**
**Trehalose**	**343.124978**	**1232.34**	**[M + H] + 342.117**	**2**
**L-Aspartate**	**134.045489**	**1264.115**	nd	**1**
**Unknown lignan #1**	**415.214999**	**53.372**	**[M + H] + 414.211**	**3**
**Trp/anthranilate-derived alkaloid #2**	**160.077541**	**564.5435**	nd	**3**
**L-Tyrosine**	**182.083331**	**728.615**	nd	**1**
**Methyl-propenyl-ketone fragment**	**85.0595623**	**65.906**	nd	**3**
**L-Citrulline**	**176.103539**	**1066.4**	**[M + H] + 175.097**	**2**
	159.07823	1067.43	[M + H − NH3] + 175.097	
**2-*O*-methyladenosine**	**282.121178**	**772.818**	**[M + H] + 281.114**	**2**
	150.079173	772.818	[M + H − C5H8O4] + 281.114	
**Betalamic acid**	**210.040691**	**72.512**	**[M − H] − 211.048**	**2**
	166.051461		[M − H − CO2] − 211.048	
	122.061824		[M − H − CO2] − 167.059	
	112.986128		[M − H] − 113.994	
	104.051649		[M − H − H2O] − 123.07	
	68.9973087		[M − H − CO2] − 113.994	
**Unknown lignan #2**	**415.144229**	**595.9305**	**[M − H] − 416.153**	**3**
	397.134394		[M − H − H2O] − 416.153	
**Heptaprenyl diphosphate tentative**	**675.359228**	**595.9265**	**[M − H + Na] −**	3
	653.375205		[M − H] −	
	277.21			
	101.029			
**N-Methylanthranilate**	**150.056863**	**189.953**	nd	**2**
**Malic acid**	**89.0257836**	**664.706**	nd	**1**
**UDP glucose tentative**	**565.047354**	**1328.19**	**[M − H] − 566.053**	**2**
**Monodiacylglycerol**	**775.536748**	**176.006**	**[M + Na + ACN] −**	**3**
	712.537		[M − H] −	
	683.3995			
	661.419			
	550.4766		[M − Hexose] −	
	532.4626		[M-glucose] −	
**Unknown Carboxylic acid**	**310.073376**	**639.003**	**[M − H] −**	**3**
	266.083214		[M − H − CO2] − 311.081	
**Methanesulfonic acid**	**94.9826702**	**401.101**	nd	**2**
**L-Erythrulose**	**119.036098**	**704.597**	**[M − H] −**	**2**
	101.025423		[M − H − H2O] −	
	71.0160475			
	59.0163848			
**Glyceric acid**	**105.020724**	**879.954**	nd	**2**
	75.03			

Table S5. Nucleotide sequence of primers used for qRT-PCR analysis.

Gene	Name	Gene Model	Forward Primer (5´→3´)	Reverse Primer (5´→3´)	Reference
DREB2	Dehydration-responsive element-binding protein 2 (DREB2)	Solyc05g052410.1.1	GCAAGAGGACTTCCACTTCT	GCCATGTTGCCAATGCACCAA	[37]
RAB18	Responsive to ABA 18	Solyc02g084850.2.1	CCTGGGATGCATTGAACACC	CACGGGACACCATAACACAC	[38]
NCDE1	9-cis-epoxycarotenoid dioxygenase 1 (NCED1)	Solyc07g056570.1.1	CTTATTTGGCTATCGCTGAACC	CCTCCAACTTCAAACTCATTGC	[46]
AOC	Allene oxide cyclase	Solyc02g085730.2.1	GCCTCTGCTGCTCTTAGAACC	CGAAGATAAGCAGGGCTTCC	[46]
JA2	Jasmonic acid 2	Solyc12g013620.1.1	GCCCATCCTCCAAATTTCCG	CTACTGCTTGAACCCGAGATT	[40]
PR-1a	Pathogenesis-related protein 1a	Solyc09g007010.1.1	TGGTGGTTCATTTCTTGCAACTAC	ATCAATCCGATCCACTTATCATTTA	[22]
MYC2	MYC2	Solyc08g076930.1.1	CGGTGTCATCACCTGCTTAT	TTCGGTGTCGGTAACTTCTTC	[21]
Actin	Actin	Solyc03g078400.2.1	CCTCAGCACATTCCAGCAG	CCACCAAACTTCTCCATCCC	Isabel Diaz unpublished results (CBGP, Madrid Spain)
EF1a	Elongation factor 1-alpha	Solyc06g009970.2.1	GACAGGCGTTCAGGTAAGGA	GGGTATTCAGCAAAGGTCTC	[38]
